# Sporadic Burkitt Lymphoma Presenting with Middle Cranial Fossa Masses with Sphenoid Bony Invasion and Acute Pancreatitis in a Child

**DOI:** 10.1155/2021/6610666

**Published:** 2021-09-14

**Authors:** Tal Dror, Virginia Donovan, Naomi Strubel, Sucharita Bhaumik

**Affiliations:** ^1^Department of Pediatrics, NYU Langone Hospital—Long Island, 259 First Street, Mineola, NY 11501, USA; ^2^Department of Pathology, NYU Langone Hospital—Long Island, 259 First Street, Mineola, NY 11501, USA; ^3^Department of Radiology, NYU Langone Hospital—Long Island, 259 First Street, Mineola, NY 11501, USA; ^4^Department of Pediatric Hematology/Oncology, Cancer Center for Kids, NYU Langone Hospital—Long Island, 120 Mineola Blvd. Suite 460, Mineola, NY 11501, USA

## Abstract

Acute pancreatitis in children is usually due to infection, trauma, or anatomical abnormalities and is rarely due to obstruction from malignancy. Sporadic Burkitt lymphoma (BL) is an aggressive non-Hodgkin B-cell lymphoma that usually involves the bowel or pelvis, with isolated cases presenting as acute pancreatitis. We report a case of BL in a 12-year-old male presenting as acute pancreatitis with obstructive jaundice and a right middle cranial fossa mass invading the sphenoid bone. The common bile duct in this case was dilated to 21 mm in diameter on abdominal ultrasound and to 26 mm on magnetic resonance cholangiopancreatography (MRCP), significantly greater than any value reported in the literature for BL. Given the rapidly progressing nature of BL, we emphasize the importance of recognizing heterogeneous presentations of this disease to improve patient survival. We also conclude that it is important to consider malignancy in a child with acute pancreatitis, particularly in the presence of obstructive jaundice or multisystem involvement. *Other Presentations*. This case report has no prior publications apart from the abstract being accepted to the 2020 SIOP (International Society of Pediatric Oncology) meeting and 2020 ASPHO conference (canceled due to the COVID-19 pandemic) and subsequently published as an abstract only in *Pediatric Blood and Cancer*. We have also presented the abstract as a poster presentation at our institution's (NYU Langone Hospital—Long Island, previously known as NYU Winthrop) annual research day conference in 2020.

## 1. Introduction

Acute pancreatitis in children is mostly due to infection, trauma, anatomical abnormalities, or medications and is rarely due to obstruction from malignancy [1]. Few cases of non-Hodgkin lymphoma in adults and even less in children have been reported to cause acute pancreatitis with obstructive jaundice through invasion of the head of the pancreas and compression of the pancreatic and common bile ducts [1, 2].

Burkitt's lymphoma (BL) is an aggressive rapidly dividing non-Hodgkin B-cell lymphoma that often presents with extranodal masses. It comes in three variants—endemic, sporadic, and immunodeficiency-associated subtypes. The sporadic variant is mostly seen in the United States and Western Europe. This form of BL accounts for 30% of pediatric lymphomas and usually involves the abdomen (generally the bowel or pelvis), unlike the endemic subtype seen in Equatorial Africa and New Guinea, which presents with a facial mass [3].

We describe a case of a twelve-year-old boy who presented with severe pancreatitis associated with obstructive jaundice and a middle cranial fossa mass invading the sphenoid bone, who was diagnosed with Burkitt's lymphoma. The common bile duct (CBD) was dilated up to 2.6 cm on magnetic resonance cholangiopancreatography (MRCP), significantly higher than any value reported in the literature for Burkitt's lymphoma. This case highlights the importance of considering malignancy in a child with acute pancreatitis, especially in the presence of multisystem involvement.

## 2. Case Report

A previously healthy twelve-year-old male presented to the emergency department (ED) at NYU Langone Hospital - Long Island with abdominal pain, jaundice, and a right-sided facial mass. Prior to arrival, the patient had two weeks of bilateral knee pain, followed by right upper quadrant abdominal pain, multiple episodes of nonbloody nonbilious emesis, and weigh loss. There were no fevers, night sweats, sore throat, or travel abroad. During this time, he also developed right-sided facial swelling in the preauricular area that was initially tender and then numb with time. The patient was taken to an outside hospital where he was admitted for three days for transaminitis and acute pancreatitis. Amylase and lipase levels were 305 U/L and >1000 U/L, respectively. The abdominal ultrasound conducted there was normal. Labs improved with supportive care and the patient was discharged home.

The following day, the emesis resumed. Abdominal X-ray performed by the pediatric gastroenterologist showed moderate to severe constipation and polyethylene glycol was started. Three days later, the patient developed jaundice and was taken to the NYU Langone Hospital—Long Island ED for further evaluation.

**Figure 1:**
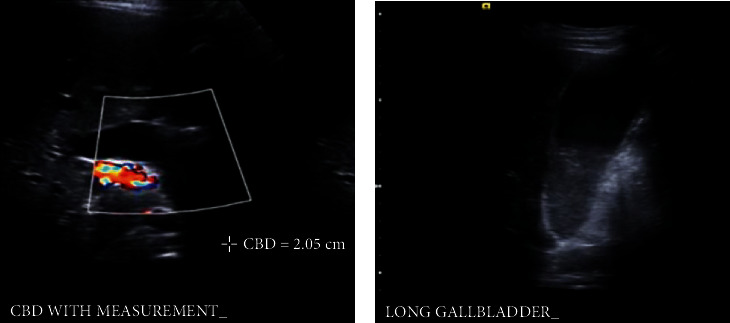
Abdominal ultrasound revealing dilated common bile duct measuring (a) 2.05 cm and gall bladder with (b) biliary sludge.

**Figure 2:**
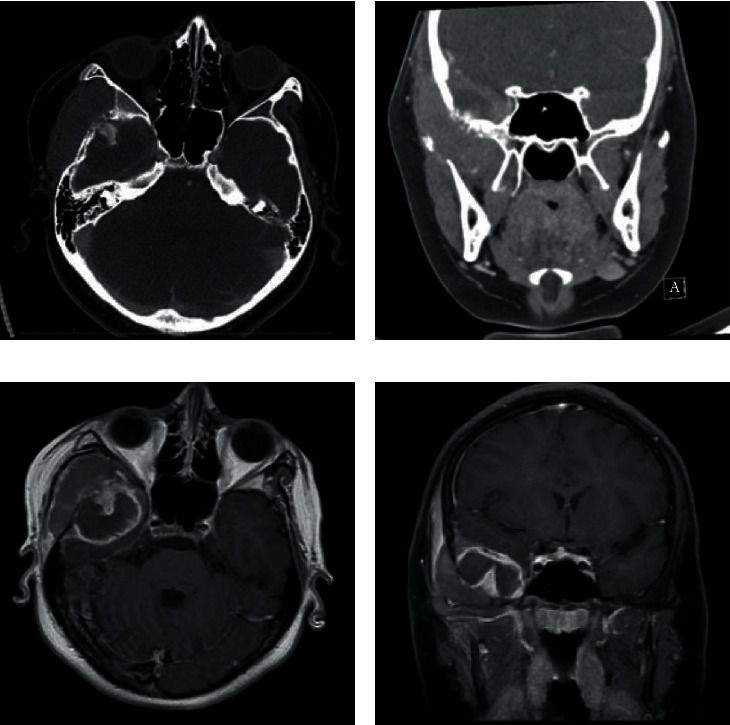
Lytic changes and destruction of sphenoid bone with adjacent right middle cranial fossa mass. (a) Head CT: bone window focusing on the destruction of the right sphenoid wing. (b) Head CT: brain window focusing on 3.0 × 4.4 × 2.4 cm peripherally enhancing mass in the right middle cranial fossa. (c, d) Brain MRI showing 2 × 2.9 × 5.2 cm right temporal region mass and irregularity under the right sphenoid wing and an adjacent 4.5 × 3.1 × 2.5 cm intracranial mass.

In the ED, liver enzymes were elevated with an alanine aminotransferase (ALT) of 249 U/L, aspartate aminotransferase (AST) of 142 U/L, and alkaline phosphatase (ALP) of 361 U/L. Signs of biliary obstruction were evident with a gamma-glutamyl transferase (GGT) of 335 U/L, total bilirubin of 6.8 mg/dL, and direct bilirubin of 4.6 mg/dL. Lactate dehydrogenase (LDH) was 402 U/L. Inflammatory markers were also increased with a C-reactive protein (CRP) of 132 and an erythrocyte sedimentation rate (ESR) of 50. The respiratory viral panel was negative. Epstein-Barr virus titers were indicative of past infection. Chest X-ray was normal. Abdominal ultrasound showed a distended gallbladder with sludge and stones and a dilated common bile duct measuring 21 mm (Figure 1). Computerized tomography (CT) of the facial bones revealed an abnormal right sphenoid wing with lytic changes, destruction, and a medially adjacent 3.0 × 4.4 × 2.4 cm peripherally enhancing lesion in the right middle cranial fossa (Figures 2(a) and 2(b)). The right temporalis muscle was displaced by the mass and there were lucent changes in the adjacent left zygomatic arch and widening of the right temporomandibular joint. Given radiologic and laboratory findings, the patient was made nothing per os (NPO), started on piperacillin tazobactam and intravenous fluids, and admitted to the pediatric floor.

Magnetic resonance cholangiopancreatography (MRCP) done on hospital day one revealed marked dilation of the common bile duct (CBD) to 26 mm, without associated cholelithiasis or cholecystitis. There was mild splenomegaly measuring 13.3 cm and signs of pancreatitis at the pancreatic head. Abdominal CT showed a dilated common bile duct with dilation extending up to the intrahepatic biliary ducts. Chest CT revealed regions of right basilar subpleural thickening versus nodules, representing an infectious, inflammatory, or metastatic process.

**Figure 3:**
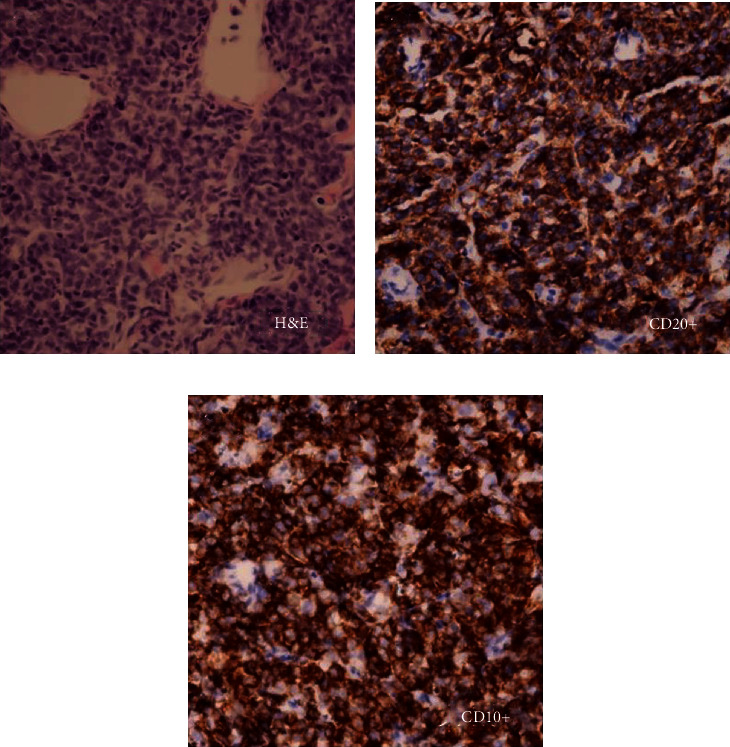
Right middle fossa biopsy showing (a) H&E stain, (b) CD20+ cells, and (c) CD10+ cells.

In parallel to the abdominal work up, brain magnetic resonance imaging (MRI) was done on hospital day one. A 6.2 × 2.9 × 5.2 cm right temporal region mass was visualized, adjacent to the temporalis muscle. There was irregularity of the right sphenoid wing with an adjacent 4.5 × 3.1 × 2.5 cm intracranial mass (Figures 2(c) and 2(d)). The mass also extended into the right temporomandibular junction with associated joint effusion being present. Prominent left retropharyngeal and posterior left neck nodules were also visualized. CT-guided biopsy of the right middle fossa mass revealed a high-grade small blue cell tumor, suggestive of non-Hodgkin lymphoma (Figure 3).

**Figure 4:**
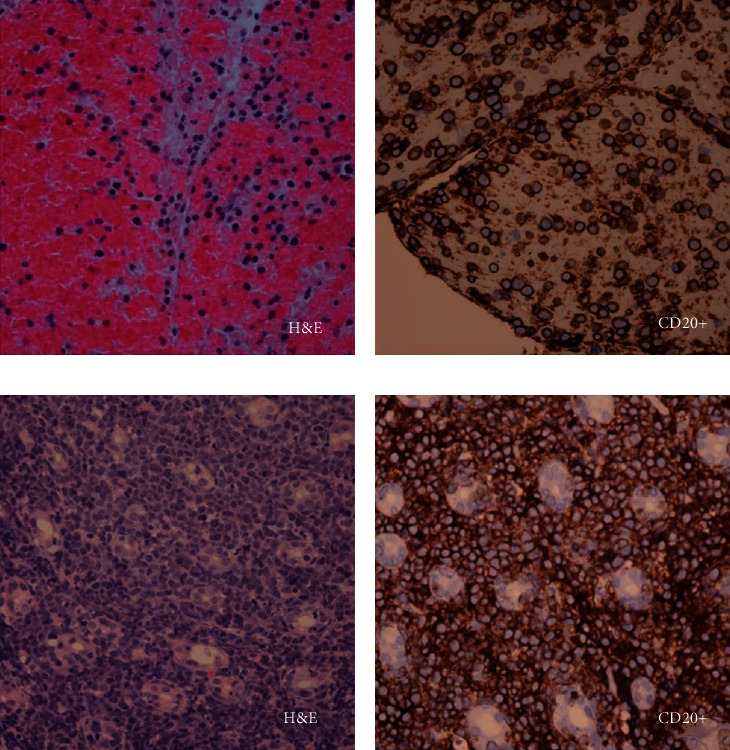
CD20-positive cells detected from pancreatic and gastric biopsies. (a, b) fine needle aspiration (FNA) of the pancreas stained with hematoxylin and eosin (H&E) and CD20. (c, d) Gastric lesion biopsy stained with H&E stain and CD20.

Endoscopic ultrasound (EUS) followed, showing biliary sludge and a 3 mm biliary stricture and a hypoechoic mass at the head of the pancreas. Fine-needle aspiration of the pancreatic mass revealed CD20(+) and CD3(−) cells, suggestive of B-cell lymphoma (Figures 4(a) and 4(b)). Biliary sphincterotomy with metal stent placement at the stricture site was then performed via endoscopic retrograde cholangiopancreatography (ERCP), draining a large quantity of the biliary sludge. Multiple gastric lesions were identified during the procedure, with biopsies revealing diffuse infiltration of lamina propria by atypical CD20+ lymphoid cells with crypt involvement (Figures 4(c) and 4(d)). IGH-MYC gene rearrangement with *t*(8,14) were detected in 75% of 200 interphase nuclei, suggestive of Burkitt's lymphoma. Pancreatitis and transaminitis improved following stent placement.

Lytic lesions were assessed via X-rays of the lower extremities. Lytic lesions were present in the right proximal tibial metaphysis and left distal femoral metaphysis, and a sclerotic lesion was identified in the right proximal tibial diaphysis; however, bilateral bone marrow aspirates were negative for IGH-MYC gene rearrangements or evidence of disease. Bone marrow biopsy and peripheral blood were also negative for disease. Intrathecal methotrexate and hydrocortisone were administered and cerebral spinal fluid drawn at the time was negative for malignant cells. Allopurinol was initiated for tumor lysis prophylaxis, labs were monitored regularly, and the patient did not develop tumor lysis syndrome.

The patient's clinical presentation was consistent with stage IV high-risk BL, given biopsy findings and intracranial involvement. He was therefore treated with chemotherapy as per the Children's Oncology Group protocol—COG ANHL1131, to which he rapidly responded.

## 3. Discussion

Burkitt's lymphoma is the fastest growing tumor, doubling every 24–48 hours, making early diagnosis and treatment critical for survival [4]. Although the sporadic form of BL seen in North America, Europe, and East Asia has a relatively low annual incidence of 2 per million children under the age of 18, the annual incidence of endemic BL is estimated to be 40–50 per million in children less than 18 [4]. In these endemic regions of Africa and Papua New Guinea where malaria and early acquisition of EBV are prevalent, BL accounts for approximately 50 percent of all childhood cancers [5] and up to 90% of all lymphomas [4]. Thus, being able to recognize the heterogeneous presentations of BL is important for patient survival.

Sporadic BL most commonly presents in the abdomen in 60–80% of cases [6], with 25% of patients having ileocecal disease manifesting as intussusception or as a right lower quadrant mass that can mimic appendicitis [7, 8]. These patients often have abdominal distension, nausea, vomiting, bowel obstruction, or gastrointestinal bleeding [4]. The central nervous system involvement occurs at presentation in 13–17% of sporadic BL cases [9]. Head and neck involvement can also be seen and generally includes the oropharynx, tonsils, or sinuses. Rarely has acute pancreatitis been reported as a presenting feature of BL [10].

In a recent case report and literature review by Lee et al. on BL presenting as acute pancreatitis, a total of 15 cases were identified [2, 10–21]. Twelve of the 15 cases were children [2, 10–18], 5/15 presented with jaundice [2, 10, 11, 15, 17], and 10/15 had diffuse pancreatic enlargement [2, 10, 11, 13–18, 20, 21]. Only 1/12 reported CBD dilation up to 9 mm on MRI, significantly less than the 26 mm dilation seen in our patient on MRCP [2]. Amylase and lipase ranged broadly from 139 to 1173 and 136 to 7500 U/L, respectively [10], encompassing the levels in our patient. Only three cases identified a tumor at the head of the pancreas [10, 12], in our knowledge, no cases have presented with acute pancreatitis and an intracranial mass as was seen in our patient.

Similarly, middle cranial fossa masses, like in our patient, are also rare in children. These masses can arise intracranially or through extracranial invasion from nasopharyngeal carcinoma, neuroblastoma, or lymphoma [22]. The vast majority of middle cranial fossa masses are meningiomas, particularly in the sphenoid wing [22]. Few cases are attributable to non-Hodgkin's lymphoma, with only some of these being due to BL [9, 22, 23]. Enlarged abdominal lymph nodes were seen in one of these cases; [9] however, there was no pancreatic involvement.

Despite its aggressive nature, BL is highly responsive to chemotherapy with an overall cure rate of 90 percent for the sporadic type in high-income countries [4]. Given the rapid doubling time of BL, it is important to recognize its diverse presentations and consider this differential in children presenting with acute pancreatitis, especially in those with obstructive jaundice and multisystem complaints.

## Data Availability

Data for this case report can be obtained from the electronic medical record and paper charts of the patient in the study upon request.
